# Fast tree aggregation for consensus hierarchical clustering

**DOI:** 10.1186/s12859-020-3453-6

**Published:** 2020-03-20

**Authors:** Audrey Hulot, Julien Chiquet, Florence Jaffrézic, Guillem Rigaill

**Affiliations:** 1 Université Paris-Saclay, INRAE, AgroParisTech, GABI, Jouy-en-Josas, 78350 France; 2 Université Paris-Saclay, AgroParisTech, INRAE, UMR MIA-Paris, Paris, 75005 France; 3 Université Paris-Saclay, UVSQ, Inserm, Infection et inflammation, Montigny-Le-Bretonneux, 78180 France; 4 Université Paris-Saclay, CNRS, INRAE, Univ Evry, Institute of Plant Sciences Paris-Saclay (IPS2), Orsay, 91405 France; 5 Université de Paris, CNRS, INRAE, Institute of Plant Sciences Paris-Saclay (IPS2), Orsay, 91405 France; 6 Université Paris-Saclay, CNRS, Univ Evry, Laboratoire de Mathématiques et Modélisation d’Evry, Evry, 91037 France

**Keywords:** Hierarchical clustering, Data integration, Unsupervised learning, Consensus clustering, Omics

## Abstract

**Background:**

In unsupervised learning and clustering, data integration from different sources and types is a difficult question discussed in several research areas. For instance in omics analysis, dozen of clustering methods have been developed in the past decade. When a single source of data is at play, hierarchical clustering (HC) is extremely popular, as a tree structure is highly interpretable and arguably more informative than just a partition of the data. However, applying blindly HC to multiple sources of data raises computational and interpretation issues.

**Results:**

We propose *mergeTrees*, a method that aggregates a set of trees with the same leaves to create a consensus tree. In our consensus tree, a cluster at height *h* contains the individuals that are in the same cluster for all the trees at height *h*. The method is exact and proven to be $\mathcal {O}(nq\log (n))$, *n* being the individuals and *q* being the number of trees to aggregate. Our implementation is extremely effective on simulations, allowing us to process many large trees at a time. We also rely on *mergeTrees* to perform the cluster analysis of two real -omics data sets, introducing a spectral variant as an efficient and robust by-product.

**Conclusions:**

Our tree aggregation method can be used in conjunction with hierarchical clustering to perform efficient cluster analysis. This approach was found to be robust to the absence of clustering information in some of the data sets as well as an increased variability within true clusters. The method is implemented in R/C++ and available as an R package named mergeTrees, which makes it easy to integrate in existing or new pipelines in several research areas.

## Background

Data integration has become a major challenge in the past decade as an increasing amount of data is being generated from diverse sources, leading to heterogeneous and possibly high-dimensional data. It is thus essential to develop new methods to analyze multiple data sets at the same time, by taking into account the relationships between the sources and the different underlying mechanisms originating the data. This paper is part of this scope by introducing unsupervised tools to explore multiple hierarchies, built from heterogeneous and multi-source data, typically found in the omics field.

With omics, many studies were successful for linking a particular phenotypic trait to one kind of omic features [1, 2]. However, multi-omics data is the new standard, since integrating several sources (genotyping, transcriptomics, proteomics, and more) is needed to have a finer understanding of the biological processes underlying the phenotypes. Typically, having a better omics-characterization of a disease could help to adjust the prediction of the outcome and the treatment of the patients. Therefore, multi-omics data analyses have recently received much interest in medical research [3, 4].

Unsupervised methods – and in particular clustering – are routinely used in omics in order to discern grouping patterns between the observations and link the groups to an outcome such as death or disease. Hierarchical clustering (HC) builds an attractive tree structure with a simple interpretation and is therefore a method of choice in exploratory analyses. Indeed, HC allows to efficiently visualize group structures in the data for various numbers of groups. However, it is not directly adapted to the analysis of multiple, heterogeneous data sources.

In this paper, we propose a novel method and compare it to two existing ones for recovering a single hierarchy – or tree structure – between individuals for which multiple sources of data are available. Although the most natural way to reach this goal is to merge the data sets or the dissimilarities before applying HC, we propose a method that aggregates the result of several HC into a single hierarchy. To this end we introduce a fast tree aggregation algorithm that can deal with many hierarchies to merge. The overall complexity of our tree aggregation method is $\mathcal {O}(nq\log n)$, with *q* being the number of sources and *n* the number of individuals.

The rest of the paper is organized as follows: first, we give an overview of the methods that address a similar problem in the literature, in different yet related communities (machine learning, phylogenetics, bioinformatics). This leads us to introduce the rationale for developing our own method for recovering a single hierarchy from multiple data sets, that we describe in the next section. In particular, we detail the algorithm that aggregates multiple tree structures with a low computational burden. Numerical and statistical performances of the aggregation methods are then studied on simulations. Finally, we illustrate our method on two multi-omics data sets, in breast cancer and cell differentiation.

### Related work

Retrieving a consensus classification out of several possible classifications is a recurring topic in many fields, such as machine learning, multi-omics and phylogenetics. In this section, we present some of the existing methods that yield a tree in these research areas and discuss the novelty of the proposed algorithm.

#### Machine learning

In machine learning, the problem of aggregating multiple hierarchies is encountered when using convex clustering with the *ℓ*_1_-norm.

Convex clustering [5, 6] is a reformulation of hierarchical clustering into a convex optimization problem. It ensures that a unique solution is found at a given regularization parameter. The form of the regularization path depends on the choice of the norm and the weights. While algorithms exist for all weights and norms [7], they are generally computationally expensive. Moreover, if the weights are not chosen appropriately, individuals can fuse at one point and split later [8].

Using the *ℓ*_1_ norm in the optimization problem leads to an improvement of the computation time and resources. In this case the method results, however, in a set of trees, one per feature, and needs a posterior treatment to obtain a consensus clustering, typically a tree aggregation method like the one we introduce hereafter.

#### Multi-omics

Many clustering methods have been specifically developed to analyse multi-omics data. Several authors provide full reviews and benchmarks [9–11]. In particular, Wang and Gu [9] suggest the following typology: *i*) direct integrative clustering, consisting in a preprocessing of the original data set before concatenation into a single data set ready for some standard clustering analysis [12, 13]; *ii*) regulatory integrative clustering, which are based on pathways [14]; *iii*) clustering of clusters, *i.e.,* methods that take clustering made on different data sets and find a consensus [15, 16].

The methods that we introduce to recover a consensus tree are related to the clustering of clusters. However, the latter does not yield a hierarchical structure as a result. To our knowledge, no consensus tree method has been developed or applied to multi-omics data analysis. Our paper seems to be the first effort in this direction.

#### Phylogenetics

In phylogenetics it is common to bootstrap sequence alignments to compute trees to assess the robustness of a tree [17]. It is also quite common to build multiple trees from different data sets (e.g. one tree per gene). Those forests of trees are often reduced to a consensus tree.

Methods that build consensus trees in phylogenetics consider the tree as a set of bipartitions (one per edge) and keep or delete bipartitions based on their occurrence frequency in the forest and/or their compatibility with previously selected bipartitions.

Adams [18, 19] was the first to address the problem, and proposed to build a consensus tree by keeping bipartitions present in all trees of the forest. Margush and McMorris [20] relaxed the constraint by including all bipartitions present in at least half of the trees, leading to the majority rule consensus. Both of these methods suffer from conservatism and lead to polytomies in the tree. Finally Barthélémy and McMorris [21] introduced the median tree, which has an algorithmic complexity of *O*(*n*^3^) and may not be unique.

All these methods consider only the tree topology, not the branching times. In HC fusion heights are an indication of the distance between clusters and are therefore important for the statistical interpretation of the tree.

In the rest of the paper, we stick to methods yielding a single consensus tree, with at most a quadratic complexity, and relying on mathematical distances for the branching pattern.

## Methods

In this section we present our method for aggregating trees, and give the details of two other natural methods. We also investigate the complexity of these methods and different ways of applying them to get a consensus hierarchy.

### Notation

Let *X*_1_,...,*X*_*q*_ be *q* data sets, each sharing the same set of *n* individuals. For conciseness we consider that all the data sets share the same number of features *p*. Let *d* be the function used to build the dissimilarity matrix *d*(*X*) computed between all individuals of *X*. Also denote by $ \mathcal {T} = \{T_{1},..., T_{q} \}$ the set of *q* trees obtained from these data with any HC method, and by $\mathcal {C}(\mathcal {T})$ the consensus tree based on $\mathcal {T}$. The HC method used to obtain the initial set $\mathcal {T}$ does not matter. Note, however, that the tree heights should be comparable before the merge: if all the divisions in one tree *T*_*a*_ happen before the divisions of any of the other trees, then the consensus tree will be *T*_*a*_.

This raises the question of the scaling of the tables associated to each data source. Scaling is a challenge common to all methods in data integration since each source may come from different technologies or correspond to different types of signal. Therefore, they have different ranges of values and distributions (like proteomics and transcriptomics). Typically, applying HC on unscaled features can lead to a tree dominated by the table with the largest variance or range of values. In this section, we assume that the data have already been transformed so that scaling is no longer an issue. We address this question in the “Results” section when dealing with real-world data.

### Fast tree aggregation algorithm

In this section we introduce a fast algorithm called mergeTrees to build a consensus from a collection of *q* trees $\mathcal {T} = \{T_{1},..., T_{q} \}$ having the same *n* leaves. It can be summarized as follows:

For any observations *i* and *j* in {1,...,*n*}, *i*≠*j*, if *i* and *j* are not in the same cluster in at least one of the trees of $\mathcal {T}$ at height *h*, then they are not in the same cluster in $\mathcal {C}(\mathcal {T})$ at height *h*.

or, equivalently:

For any observations *i* and *j* in {1,...,*n*}, *i*≠*j*, if *i* and *j* are in the same cluster in all of the trees of $\mathcal {T}$ at height *h*, then they are in the same cluster in $\mathcal {C}(\mathcal {T})$ at height *h*.

#### Properties

The consensus tree $\mathcal {C}(\mathcal {T})$ reconstructed by mergeTrees satisfies the following properties mentioned by [22] and [23], in the phylogenetic context:

- **P1 (Anonymity).** Changing the order of the trees in $\mathcal {T}$ does not change $\mathcal {C}(\mathcal {T})$
- **P2 (Neutrality).** Changing the labels of the leaves of the trees in $\mathcal {T}$ simply relabels the leaves of $\mathcal {C}(\mathcal {T})$ in the same way.
- **P3 (Unanimity).** If the trees in $\mathcal {T}$ are all the same tree *T*, then $\mathcal {C}(\mathcal {T}) = T$

These properties ensure that we can use the method on any set of trees, as long as the trees have the same leaves and labels.

Also note that if multiple divisions occur at the same height in several binary trees, it is possible that the result is not a binary tree.

#### Algorithmic details

Our tree aggregation method proceeds in a divisive manner, by starting with all individuals in the same group and then identifying all splits of the consensus tree from the highest to the lowest. Full details of the proposed algorithm are provided in Algorithm 1 and in the following paragraph in a more intuitive manner. 
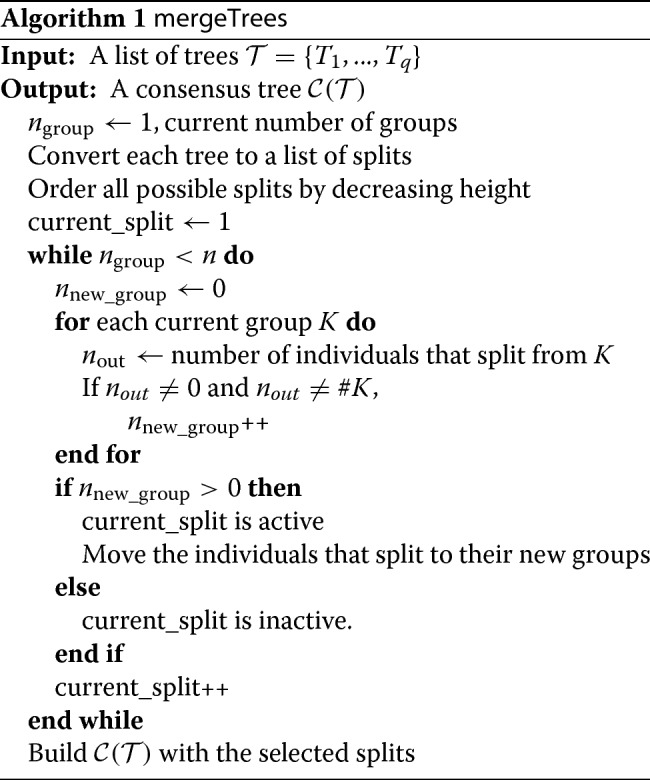


In our implementation, a tree is represented by a succession of (*n*−1) splits characterized by (*i*) the height of the split and (*ii*) the two clusters coming from this split. These two new clusters are stored as a range of indices rather than a list of indices. This is done by re-labeling in $\mathcal {O}(n)$ the leaves in such a way that the tree is ordered or plane. The algorithm takes as input *q* trees and thus (*n*−1)×*q* splits. The algorithm initializes a unique cluster with all *n* leaves. It then processes all splits from the highest to the lowest and checks whether they create a new cluster or not. A split that creates a new cluster is labelled as *active* and the group structure is updated. A split that will not impact the current group structure is labelled as *inactive*. The key idea of the algorithm is to detect active splits using only the smallest cluster of each split.

This is done with four loops over all leaves of the smallest cluster. The first loop increments the leaf group counter by one. The second loop checks whether the leaf group is active by checking whether the group counter is strictly smaller than the group size. The third loop allocates the leaf to its new group if necessary. The fourth resets the leaf group counter to zero.

**Fig. 1:**
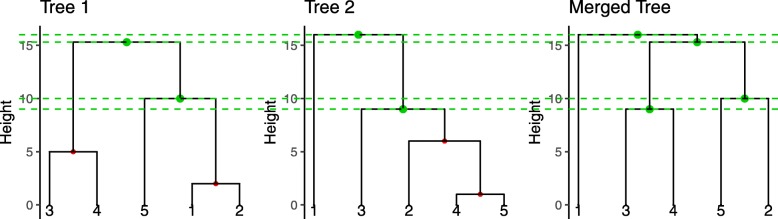
Illustration of the aggregation tree method. Aggregation of 2 trees, Tree1 (left) and Tree2 (middle) in a Merged Tree (right). The two first trees are built with 4 splits each, the consensus tree is constituted of 2 splits from the first tree and 2 splits from the second tree. Green nodes represent active splits and red nodes inactive splits

Figure 1 and Table 1 provide a toy example to illustrate how the method works. The third hierarchical clustering is the result of the merging of the first two. Green horizontal dashed lines indicate the active splits.

**Table 1:** Description of the trees in Fig. 1

	Tree	Split	Height	Cluster 1	Cluster 2	Active
1	2	1	16	1	2, 3, 4, 5	active
2	1	1	15.3	3, 4	1, 2, 5	active
3	1	2	10	5	1, 2	active
4	2	2	9	3	2, 4, 5	active
5	2	3	6	2	4, 5	inactive
6	1	3	5	3	5	inactive
7	1	4	2	1	2	inactive
8	2	4	1	1	4	inactive

Splits are ordered by overall height

#### Space complexity

The structures of the trees are stored using matrices of size *n*×3. All operations are made through vectors of length *n*. The space complexity of our algorithm is thus $\mathcal {O}(nq)$.

#### Time complexity

The complexity of Algorithm 1 to merge *q* trees with *n* leaves each can be shown to be in $\mathcal {O}(q n\log (n))$. The proof is given in Additional file 1. Intuitively the *n* log(*n*) appears because the algorithm only uses the smallest cluster of each split. This complexity allows the merging of a large number of trees with a high number of individuals/leaves.

### Methods for data integration

In the previous section the set of trees $\mathcal {T}$ is assumed to be known. Here, we include the cost of the construction of $\mathcal {T}$ from the data sets $X_{1}, \dots, X_{q}$ into the build of the final consensus tree $\mathcal {C}(\mathcal {T})$. Recall that we assume that all data sets have the same number of features *p* for clarity.

In the following, we will refer as MC (short for *mergeTrees Clustering*) for the combination of a method that yields a set of trees and the aggregation of these trees with the mergeTrees algorithm. We will focus, for now, on the use of the classical hierarchical clustering method to build the trees.

Apart from using our mergeTrees algorithm on several trees, two other natural methods come to mind. The first idea (*Direct Clustering*, in short DC) is to directly merge the data into a single table: the aggregation criterion is applied on *d*(*X*^*c*^) where *X*^*c*^=[*X*_1_,...,*X*_*q*_] is the aggregated table. The second idea (*Averaged Distance*, or AD) is to make the consensus on the dissimilarity matrices before applying HC, by averaging these matrices. Here, the aggregation criterion is applied on $\frac {1}{q} \sum ^{q}_{j = 1} d(X_{j})$.

#### Time complexity including clustering

There are two major operations to build the consensus tree in AD and DC: computation of the dissimilarity matrices and computation of the hierarchical clusterings. The computation of *q* distance matrices of size *n*×*n*, using *p* features has a complexity of $\mathcal {O}(n^{2}qp)$. This is the same complexity to create one unique *n*×*n* distance matrix out of a *n*×(*pq*) matrix. The complexity of the agglomerative step of hierarchical clustering is at least $\mathcal {O}(n^{2})$ [24].

To sum-up,

- DC is in $\mathcal {O}(n^{2}pq)$ (complexity for computing a *n*×*n* dissimilarity matrix out of a *n*×*qp* matrix and building the final HC).
- AD is in $\mathcal {O}(n^{2}pq)$ (complexity of making *q* dissimilarity matrices of dimension *n*×*n* using *p* features, averaging the matrices and building the final HC).
- MC is in $\mathcal {O}(n^{2}pq)$ (complexity of making *q* distance matrices of dimension *n*×*n* using *p* features, building all HC and aggregating them).

In MC, note that the complexity of mergeTrees is dominated by the computational cost of the *q* dissimilarity matrices. Hence, all methods have the same time complexity when using a classical way of building the hierarchical clusterings. In case of a large number of leaves, this quadratic computation is a liability and the log-linear computation time of the tree aggregation method does not lead to any advantage.

We propose in the next paragraph an approach using the mergeTrees algorithm combined with dimension reduction to reach an overall log-linear complexity.

#### Dimension reduction and improvement of time complexity

In the previous paragraph, we detailed the complexity of the mergeTrees algorithm when combined with a classical hierarchical clustering. The algorithm can be applied on any set of trees, regardless of the method used to build them. This allows to use faster approaches than HC.

In this paragraph, we introduce a way of reducing the overall time complexity of MC by considering a dimension reduction before building the trees.

For both statistical and algorithmic reasons, we suggest to perform a spectral decomposition on the concatenated data sets (i.e. one table of dimensions *n*×*pq*), taking only a small amount of the new features and to create the consensus clustering on them. Using truncated SVD (tSVD) to retrieve *k*≪*pq* axes leads to a complexity of $\mathcal {O}(npqk)$ [25]. In certain cases, using randomized SVD (rSVD) to retrieve *k* can be a better choice as the complexity of this procedure is $\mathcal {O}(npq\log k)$.

Although it makes sense to simply apply an HC algorithm on the results of the SVD, we propose a different approach. As the new features obtained by the SVD are orthogonal, each of them carries different but complementary information extracted from all the data sets. We therefore feel it makes sense to form a consensus tree out of the set of trees given by the vectors.

Combining a tSVD, a hierarchical clustering and an aggregation method leads to an overall complexity of $\mathcal {O}(kn^{2} + npqk)$. When considering the data in the form of vectors, a hierarchical clustering using Ward’s aggregation criterion and Euclidean distance can be obtained directly without computing a distance matrix. Building a tree with this method has a complexity of $\mathcal {O}(n \log (n))$ per feature, so using such an approach to build the collection of *q* trees before applying mergeTrees leads to a complexity of $\mathcal {O}(qn\log (n))$ for the MC method. Combining this method with the tSVD dimension reduction technique, the overall complexity is $\mathcal {O}(kn\log (n) + npqk)$ for MC.

Note that *kn*^2^+*npqk* is larger than *kn* log(*n*)+*npqk*, which means that MC using a spectral decomposition is faster for large *n* and *k* small enough.

This direct way of obtaining a clustering in $\mathcal {O}(n\log (n))$ is not possible for DC and AD methods. Indeed, AD relies on the computation of the distance matrices, and DC concatenates all features available into a unique matrix. DC on the spectral vectors is actually the result of a hierarchical clustering performed on the tSVD decomposition of the concatenated data sets.

We will call spAD, spDC and spMC the spectral variants of the methods, i.e. the methods applied on the vectors of an SVD decomposition.

#### Timing simulations.

**Fig. 2:**
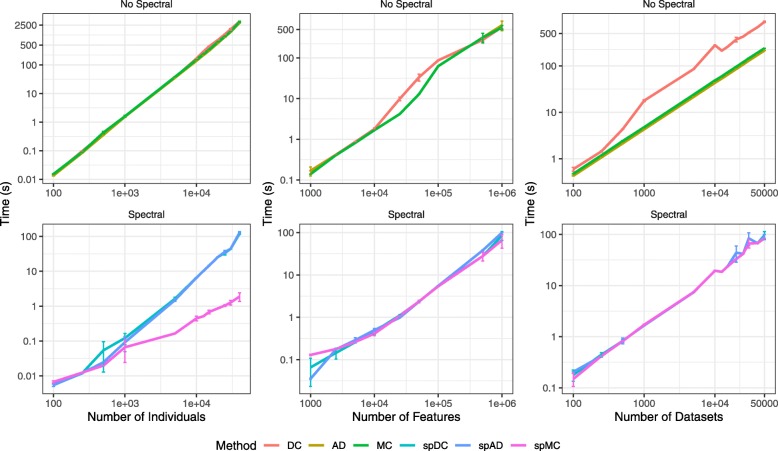
Timing simulations for the different methods. Number of tables, individuals and features are set to 3, 100 and 100, respectively, when they are not the variable of interest. For the spectral methods, 3 axes were computed by random SVD

Results for timing simulations are shown in Fig. 2.

Timing simulations were performed for all methods and their spectral alternatives. They were repeated three times and averaged. The influence of the number of individuals per data set, the number of features and the number of data sets was studied. In the first simulation design, the number of features per table was set to 100 with 3 tables, and the number of individuals was increased up to a very large number. The opposite design was used for the second simulation scheme, with the number of features set to 100 with 3 tables, and the number of individuals increasing. For the last simulation, the number of individuals and features were set to 100 and the number of tables available increased. For all the spectral applications, *k*=3 axes were computed with randomized SVD. The time needed for concatenating all data sets into one before applying the rSVD procedure is included in the time dislayed in the spectral panels.

The three methods in the non spectral case have the same complexity, which is verified in the graphs for the individuals and features per table, as the three curves have the same trend. DC was found to be the most time consuming when dealing with a lot of data sets. The step of computing the distance out of the concatenation result causes an increase in the total time.

When increasing the number of individuals, spMC clearly outperforms its competitors by several orders of magnitude.

The spectral decomposition allowed to considerably reduce the computing time required for all the methods, especially in the case of large numbers of individuals.

#### Implementation

We implemented the mergeTrees algorithm in an R/C++ package called **mergeTrees** available on CRAN [26]. In our analyses, we always rely on Ward’s hierarchical clustering and Euclidean distances. With multivariate data, we use the implementation available in the R-base function *hclust* [27]. With vector data, we use the $\mathcal {O}(n \log (n))$ implementation available in the ward_1d function of the package **univarclust** [28].

## Results

### Simulation study

To compare the performance of AD, DC, MC and their spectral variants (spAD, spDC and spMC), we generated 5 tables of *n*=125 individuals and *p*=150 features. Tables were generated vector by vector, $\{\mathbf {y}_{j}, j=1,\dots p \}$ so that $\mathbf {y}_{j} = (y_{1j}, \dots, y_{nj})^{\intercal } \in \mathbb {R}^{n}$ are realizations of Gaussian variables defined by

$$ Y_{ij} = \left\{\begin{array}{ll} \mu_{i(k)} + \varepsilon_{ij}, & \text{ for}\ j=1,\dots,50\\ \varepsilon_{ij},& \text{ for}\ j=1,\dots,100 \end{array}\right. $$

where $\varepsilon _{ij} \sim \mathcal {N}(0, 1)$. Hence, only the first 50 features of each table carry some information about a group structure defined by the means *μ*_*i*(*k*)_ as follows: the *n* individuals are divided into *K*=5 balanced groups so that *μ*_*i*(*k*)_=*s*×*k* with *i*(*k*) the group of individual *i*, and *s* a separability factor. This separability factor is introduced to control the difficulty of retrieving the underlying classifications of the individuals: a larger separability factor means more distant groups, while the within-group variance remains the same. Two scenarios are considered: one where all informative features describe the 5 groups, and one where the group information is split among the tables (only 2 groups are represented in each table). For the spectral variants, the feature vectors are bound into one data set on which the SVD is performed. Two axes are retained to form the new set of feature vectors on which AD, DC or MC are applied.

To compare the accuracy of the different methods, we rely on the *Normalized Information Distance* (NID) [29], a distance between partitions based on mutual information. A value of 1 means two partitions with nothing in common, while a distance of 0 means identical partitions. The NID is computed for 5 repetitions of the experiment and averaged, at each level of the reconstructed trees.

**Fig. 3:**
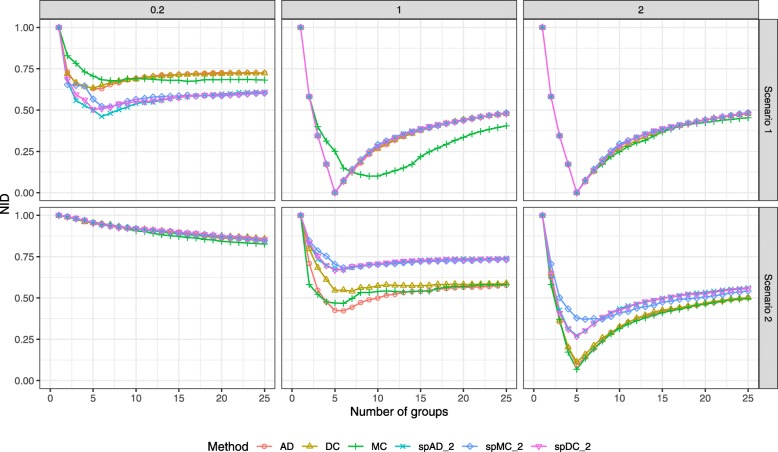
Simulation results for different separability factors and two scenarios. Scenario 1 (top row): each informative vector contains information on the 5 groups. Scenario 2 (bottom row): each informative vector contains information about 2 of the 5 groups. Columns represent the separability factor between the groups, from the most difficult situation to the easiest one

Figure 3 shows the results of the simulations. The same pattern is observed in both scenarios: when the separability factor is low, all methods struggle to find the correct classification. As the separability increases, the NID is minimized for the true number of groups (*k*=5) for most of the methods. The spectral alternative improves the results for MC when considering the first scenario.

In the second scenario, where the group information is spread among the informative features, the non-spectral alternatives perform better. Having two groups per table allows a better differentiation of the groups, hence, each data set provides a more precise classification, which is reflected on the consensus trees. However, even when the separability is high, the spectral alternatives have trouble finding the classification.

### Multi-omics data

To illustrate our approach in the context of unsupervised analysis of real-world data with multiple tables, we consider two multi-omics data sets from breast cancer and cell differentiation.

In order to avoid differences in the distances and heights of the trees that would hamper the tree aggregation process, each table are centered and standardized by means of its maximum singular value. The spectral decomposition was performed on the modified data sets, and the new features were neither scaled or centered. Hierarchical clusterings were first built on the separate tables to show the NID values obtained when considering only one type of data. Then the three methods presented above: Direct clustering (DC), Averaged distance (AD) and the proposed mergeTrees Clustering algorithm (MC) were applied, as well as their spectral versions. For AD and DC, the distance matrices and trees are calculated on each table separately, then aggregated.

For each of the obtained trees, we retrieve the classification they provide at each level of division, and compare them to one or more clinical outcomes, using the NID. The results we present in this section are the minimum NID values found and the associated number of groups.

#### Cell-type differentiation

The first data set concerns the inflammatory bowel disease and is presented by Ventham *et al.* [30]. Methylation (485577 features) and gene expression (46835 features) data were available for 199 samples. Different cell-types were considered: CD14 (57 samples), CD4 (51 samples), CD8 (47 samples) and whole blood (44 samples) were sequenced, originating from 61 individuals. All methylation and gene expression data are freely available at NCBI GEO database [31] (accession GSE87650). Individual clusterings based on the methylation and gene expression data show that the observations tend to cluster based on the cell-type of the sample. We therefore compared the results of the three clustering methods to the cell-type repartition of the samples.

**Fig. 4:**
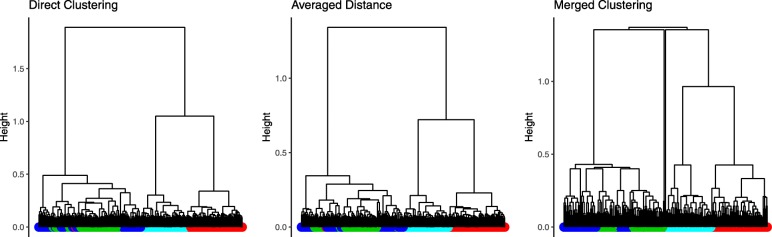
Celltype data sets. Tree results for the three multivariate non spectral methods for the cell-type data set. Colors at the bottom correspond to the leaves cell-type. Red: CD14, Green: CD4, Blue: CD8, Cyan: whole blood

Results are presented in Table 2 and in Fig. 4. Gene expression data obviously contains signal largely related to the cell-type information, since HC leads to a NID value of 0.14. On the contrary, methylation data only reaches a NID of 0.47. The spectral decomposition of the separate tables, retaining 3 axes for each, do not improve the classification.

**Table 2:** NID values and number of groups, results for the cell-type data set, taking 3 axes for the spectral decomposition

	Nb Groups	NID
**Data sets**		
Gene expression	**4**	**0.14**
Methylation	4	0.47
**Spectral axes**		
Gene expression-sp	**4**	**0.16**
Methylation-sp	6	0.53
**Multivariate methods**		
AD	4	0.27
DC	4	0.27
MC	**6**	**0.22**
**Spectral methods**		
SpAD	4	0.27
SpDC	**4**	**0.26**
SpMC	3	0.29

When analyzing the two data sets together with AD, DC and MC, all methods perform in a similar way.

Regarding the NID value, MC seems to be less impacted by the lack of information concerning the cell-type classification in the methylation data. It, however, selects more groups than expected.

The three spectral variants of the methods perform in a similar way as well. It is worth mentioning that the spectral approach helps MC to select a number of groups closer to the ground truth (from 6 to 3 groups), although the NID is higher. Overall, the three methods seem to be quite robust to this difficult case.

Figure 4 shows the trees obtained from the three non-spectral methods. The color bar at the bottom of each dendrogram represents the cell-type of the leave. MC leads to a non binary tree in this case. All the methods seem to have trouble finding the difference between CD4 (green leaves) and CD8 (blue leaves) samples.

It has to be pointed out that the consensus methods provide better NID results than the methylation data but are less efficient than the gene expression data alone. This example shows very well the behaviour of the methods when integrating data sets that are carrying different information. However, this raises the question of the choice of the data sets to be jointly analyzed to be biologically relevant.

#### TCGA multi-omics breast cancer data

The data used in this section was downloaded from the TCGA website. It consists in four types of omics to be integrated for 104 patients: methylation (21123 features), miRNA expression (725 features), protein expression (156 features), gene expression (RNA-seq, 19738 features). The RNA-seq table was log-transformed.

Clinical features such as the age at diagnosis, cancer status, cancer subtype, oestrogen and progesterone receptor status (designated by ER and PR status respectively, in the following paragraphs) are available for all patients with no missing value. The individuals (*n*=104) are patients with breast cancer distributed into four existing subtypes: Luminal A (*n*=44), Luminal B (*n*=20), HER2-enriched (*n*=18) and Basal-like (*n*=22). These subtypes are related to the ER and PR status, as the luminal subtypes are associated with positive ER and PR, and the two others are related to negative ER and PR. Clustering was first performed for each dataset separately. These individual clusterings were not found to be related to age or stage of the cancer. The protein and RNA-seq analyses reflected the ER/PR status the best. We therefore compared the results of the consensus methods to these clinical variables in order to quantify their medical relevance. The subtype was also included, as it is related to the ER/PR status and is often of interest in such studies. Results are shown in Table 3.

**Table 3:** NID values and number of groups, results for the TCGA breast cancer dataset, taking 5 axes for the spectral decomposition

	ER status		PR status		Subtype	
	NID	N	NID	N	NID	N
**Data sets**						
methyl	0.77	3	0.78	4	0.69	9
mirna	0.72	2	0.71	2	0.67	4
protein	**0.32**	**2**	**0.45**	**2**	**0.53**	**5**
rna	0.40	2	0.55	2	0.59	4
**Spectral DataSets**						
methyl-sp	0.78	3	0.84	3	0.70	6
mirna-sp	0.66	2	0.70	2	0.64	5
**protein-sp**	**0.46**	**2**	**0.48**	**2**	0.58	4
rna-sp	0.71	2	0.73	2	**0.44**	**4**
**Non spectral consensus**						
AD	0.61	2	0.66	2	**0.54**	**4**
DC	0.68	2	0.70	2	0.57	4
MC	**0.40**	**2**	**0.51**	**3**	0.56	8
**Spectral consensus**						
SpAD	0.60	2	0.61	2	0.49	4
SpDC	0.46	2	**0.54**	**2**	**0.43**	**4**
SpMC	**0.40**	**2**	0.55	2	0.56	5

Regarding the NID values based on the individual clusterings at the top of Table 3, the protein expression dataset is the most informative in the task of retrieving the ER/PR status, as well as the cancer subtype. The RNA-seq data perform nearly as well, whereas the methylation and miRNA data provide very little information with regard to these clinical variables. When considering the spectral variants, there is an increase in the performance of the RNA-seq data for the subtype classification while it decreases for the ER and PR status. On the other hand, miRNA performance is slightly improved for the ER status. Other data sets do not seem to have improved performance for any of the three clinical variables after a spectral decomposition.

When combining all these data within a multi-omics clustering approach (second part of Table 3), all the methods perform better than the methylation or miRNA data alone. They, however, often perform worse than the most informative individual table, i.e. protein. They are closer to the RNA-seq results. The proposed method (MC) for finding a consensus tree performs well to retrieve the ER/PR status, and has better performances for that purpose than the two others. AD performs better for finding a consensus for the subtype classification. MC has a close result for the NID on the subtype, but identifies 8 groups instead of 4.

The spectral analyses show a similar pattern in the results. The NID values for the MC approach remain nearly the same, but the number of groups found for the subtype with the spectral version is now equal to 5. The DC performances are improved in the spectral setting, as well as the AD approach concerning the subtype.

**Fig. 5:**
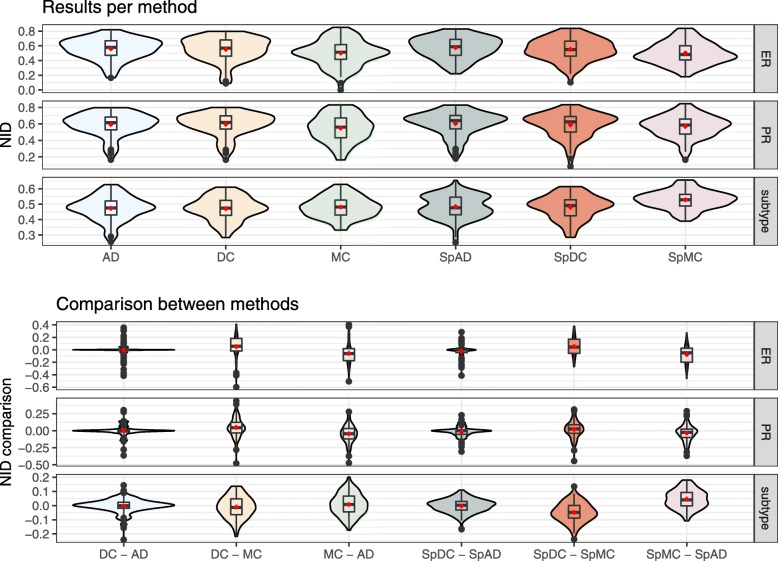
Breast Cancer data sets. Violin plots of bootstrap results (100 iterations, 80% individuals). Minimum NID obtained for each bootstrap run, without taking into account the number of groups, and differences between these minimum NID values. Spectral method results were obtained with truncated svd taking the first 5 axes of the decomposition

The stability of the methods was assessed by generating 100 subsamples with a 0.8 proportion in each subsample. Results are shown in Fig. 5. For each of them, the three methods and their spectral variants are applied and the minimum NID values were computed. The first panel shows the minimum value for each method, the second panel shows the difference of these values between the methods. All violin plots illustrate the high variablity of the results, i.e. the classification is highly dependent on the individuals chosen in the subsamples.

For the standard version of the methods, the violin plots show that DC and AD lead to similar results for the three classifications. MC leads to lower NID values for ER and PR but higher for subtype, when compared to the two others.

When considering the spectral approaches, there is an improvement for MC for the ER classification. However, MC and DC do not seem to benefit as much as AD from the spectral decomposition. Comparison of the methods shows that SpDC and SpAD perform in a similar way for the ER and PR status. SpAD is better at retrieving the subtype. SpMC seems to yield higher NID values for the subtype than the two others but lower for ER and PR status.

## Discussion and conclusion

The joint analysis of multi-omics data is a challenging research question. We presented in this paper an algorithm for aggregating multiple hierarchical trees to obtain a consensus clustering. Several advantages of the proposed method have to be pointed out. First of all, it requires no a priori knowledge concerning the optimal number of groups.

Secondly, it is highly computationally efficient on large data sets, with a complexity of $\mathcal {O}(nq\log (n))$, *n* being the individuals/leaves and *q* the number of trees to aggregate. We also introduced a way of combining dimension reduction with building and aggregating the trees in a sub-quadratic overall complexity, allowing to deal with high-dimensional data. This spectral variant can help to retrieve the predominant clustering pattern of the data in a non-linear way. Finally, our approach requires very little data pre-processing, as only centering and standardization by the first singular value is necessary to ensure similar heights in the trees and proper integration. Note that the method can also be of interest when only a set of trees is known.

Several scenarios were investigated in the simulation study. We considered the case where all the features share the same classification information, and then divided the information among the features. The proposed method was compared to two other approaches that either merge all the data sets or vectors into one table, or average the distance matrices obtained on each dimension separately. As expected, the more noise was introduced in the groups, the less the methods were able to retrieve the underlying simulated classification. Our spectral alternative was able to improve the MC performances in the case where all the data sets carry the same information. Two real data sets were also analyzed. The same pattern was observed for both applications. The information contained in one data set was diluted when merged with another data set that did not have the same underlying classification. For the TCGA breast cancer data, the MC approach retrieved well the ER/PR status and performed close to the most informative individual -omics data set for these two clinical variables. In the cell-type case, the three methods performed in a similar way being impacted by the methylation data set.

To conclude, these analyses show that it is important that the data tables integrated in multi-source data provide coherent information to deliver a meaningful global analysis. In the case of contradictory information, it is difficult to automatically merge these data without hampering the interpretation. Nevertheless, our data integration approach is robust to the presence of data tables that do not carry any information.

An interesting research direction is to use the consensus tree approach to compare a set of hierarchical clusterings sharing the same leaves, for instance in a boostrap framework. Indeed, using a distance measure between classifications such as NID or *the Adjusted Rand Index* [29, 32] at each level of divisions between the individual trees and the consensus provides a quantification of the distance between the trees and their average.

## Supplementary information

**Additional file 1** Proof of time complexity of the mergeTrees procedure.

## Data Availability

The data sets used to generate the results on the breast cancer and cell-type applications were downloaded from public databases which were cited within the manuscript. All scripts used to generate simulations and application results, including figures, are available at https://github.com/AudreH/mergeTrees/tree/article_branch/inst/article.
