# Supplementary material for: Fast tree aggregation for consensus hierarchical clustering
Source: BMC Bioinformatics. 2020 Mar 20;21:120. doi: 10.1186/s12859-020-3453-6 (PMC7085155; doi:10.1186/s12859-020-3453-6)
Supplement: Supplementary file 1 — Additional file 1 Proof of time complexity of the mergeTrees procedure. [file 12859_2020_3453_MOESM1_ESM.pdf]

Additional File 1 — Proof of time complexity of the **mergeTrees** Procedure.

Proof. We aim to prove a recurrence relation on n, the number of individuals in the data. Let $T(n)$ be the worst computation time for a $n-$tree, with the convention $T(1) = 0$. We make the assumption that we only consider, at each step, the individuals for the smallest cluster created by the division (i.e. $n/2$ individuals at most).

Then,

- $T(2) = 1$ (only one split and one element on the smallest cluster) ;
- $T(3) = 2$ (two successive splits, two individuals to consider) ;
- $T(4) = \max(1 + T(3), 2 + T(2)) = 4$

Building a tree with four individuals can lead to two different tree configurations: one that is balanced, and one where all the children are on the same side. This leads us to the following recurrence relation for the worst computation time:

$$T(n) = \max_{i=1}^{n/2}\{i + T(i) + T(n-i)\}$$

the first split will consider i individuals in the smallest new cluster, and the remaining complexity is the one of the two trees generated by this split.

Let us now prove that the above expression reduces to

$$T(n) = \frac{n}{2}\log_2(n), \quad \text{for all } n.$$

The initialization for this relation is already proven, as we showed that $T(4) = 4 = 2\log_2(4)$. We now assume the relation holds true at rank n, and consider a $(n+1)$-individuals tree. Then,

$$\begin{aligned} T(n+1) &= \max_{i=1}^{(n+1)/2}\{i + T(i) + T(n+1-i)\} \\ &\leqslant \max_{i=1}^{(n+1)/2} h(i), \end{aligned}$$

where, by the recurrence relation,

$$h : x \longmapsto x + \frac{x}{2}\log_2(x) + \frac{n+1-x}{2}\log_2(n+1-x).$$

We find the maximum of h by a basic study on $x \in \{1, \dots, (n+1)/2\}$. Straightforward differentiation leads to

$$h'(x) = 1 + \frac{1}{2\log(2)}\log\left(\frac{x}{n+1-x}\right),$$

which is positive when $x \geq (n+1)/5$. The maximum is then reached either in $x = 1$ or $x = \frac{n+1}{2}$. The two candidates for the maximum are thus

$$\begin{aligned} h(1) &= 1 + \frac{n}{2}\log_2(n) \\ h\left(\frac{n+1}{2}\right) &= \frac{n+1}{2}\log_2(n+1) \end{aligned}$$

Since $h(1) < 0$ and $h((n+1)/2) \geq 0$ for all n, h reaches its maximum in $(n+1)/2$ and thus

$$T(n+1) = \frac{n+1}{2}\log_2(n+1).$$

This gives us the complexity of the algorithm when we only consider the smallest created cluster. Having q trees to aggregate, we conclude that the final complexity of the mergeTrees Procedure is thus $\mathcal{O}(qn\log(n))$. □
